# Assessment of Salivary Biomarkers of Gastric Ulcer in Horses from a Clinical Perspective

**DOI:** 10.3390/ani15152251

**Published:** 2025-07-31

**Authors:** Marta Matas-Quintanilla, Lynsey Whitacre, Ignacio R. Ipharraguerre, Cándido Gutiérrez-Panizo, Ana M. Gutiérrez

**Affiliations:** 1 Department of Animal Medicine and Surgery, Regional Campus of International Excellence “Campus Mare Nostrum”, University of Murcia, Espinardo, 30100 Murcia, Spain; mrtmatas@um.es (M.M.-Q.); cguti@um.es (C.G.-P.); 2 BioZyme Inc., St. Joseph, MO 64504, USA; lwhitacre@biozymeinc.com; 3 Institute of Human Nutrition and Food Science, Division of Food Science, Faculty of Agricultural and Nutritional Sciences, University of Kiel, 24118 Kiel, Germany; ipharraguerre@foodsci.uni-kiel.de

**Keywords:** saliva, horse, gastric ulceration, quantification, diagnostic value, markers

## Abstract

This study arises from the search for non-invasive diagnostic alternatives for equine gastric ulceration (EGUS), which is prevalent, clinically variable and only confirmed by gastroscopy. The aim is to quantify five salivary biomarkers (IL1-F5, PIP, CA VI, serotransferrin, albumin) under clinical conditions by validated assays and analyse their diagnostic value. Horses were grouped in No EGUS (neither clinical signs of EGUS nor gastric lesions), EGUS non-clinical (apparently no clinical signs of EGUS but with gastric lesions), and EGUS clinical (obvious clinical signs of EGUS and with gastric lesions). The concentration of 5 analytes could be quantified using sandwich ELISA assays, with high precision (CV: 6.79–12.38%) and accuracy (>95%). Mean salivary levels of IL1-F5, CA-VI, serotransferrin and albumin were significantly higher in EGUS clinical horses compared to No EGUS horses, whereas PIP showed no statistical significance. EGUS non-clinical horses showed statistical differences with No EGUS horses for PIP and albumin. In addition, IL1-F5, CA-VI, serotransferrin and albumin showed moderate accuracy to distinguish between No EGUS and EGUS clinical horses (AUC ≥ 0.8), with sensitivity and specificity greater than 77% and 65%, respectively. Therefore, these biomarkers could be a promising starting point for screening horse that might have EGUS in practice.

## Simple Summary

This study aimed to properly quantify five markers (IL1-F5, PIP, CA VI, serotransferrin, and albumin) in horse saliva and assess their possible changes in Equine Gastric Ulcer Syndrome (EGUS) under different clinical conditions. EGUS is highly prevalent in horses, and so far, it’s only reliable antemortem diagnosis is gastroscopy. Validated immunoassays were used to measure these analytes in No EGUS horses and horses with EGUS (separated in two: those with obvious clinical signs and those with no apparent clinical signs). All horses were confirmed by gastroscopy. The five parameters could be measured with high precision and accuracy. The results showed significantly lower levels of IL1-F5, CA VI, serotransferrin, and albumin in the No EGUS horses than in EGUS clinical horses. These same markers showed moderate accuracy (AUC ≥ 0.8) to differentiate between the two health conditions. These findings suggest biomarkers for EGUS, shedding light on alternative, non-invasive ways of screening that would focus the diagnosis and even monitoring.

## 1. Introduction

Equine gastric ulcer syndrome (EGUS) has negative effects on the welfare and performance of horses [1,2], produces high costs for the industry [3,4], and unfortunately is a common disease in clinical practice [1,5]. Two forms of EGUS have been established, depending on their location: the glandular gastric disease (EGGD) and equine squamous gastric disease (ESGD) [1], which can occur individually or together [6]. The nonspecific symptomatology of the disease [7] makes diagnosis difficult, with gastroscopy as the only reliable antemortem diagnostic tool [8].

In recent years, efforts to find new options for EGUS diagnosis include the identification of new biomarkers using different proteomics approaches [9]. In saliva samples, proteomics studies have proposed several biomarkers for detecting gastric ulcers in horses [10,11]; however, no validations have been performed, and therefore, they are not currently available for clinical use.

Recently, we have identified and validated nine salivary biomarkers of gastric ulcers, using gel-proteomics and Western blotting, that were increased in horses with experimentally induced gastric ulcers [12], paving the way for potential detection of this highly prevalent disorder. These biomarkers are closely associated with immune (interleukin 1-F5 (IL1-F5), IL1-F6, prolactin inducible protein (PIP)) and inflammatory responses (alpha-1 antitrypsin (AAT), apolipoprotein-A4 (ApoA4), serotransferrin, albumin), and some of them also have more specific links to digestive diseases and disorders (ATT, Apo-A4, albumin, carbonic anhydrase VI (CA-VI), fatty acid-binding protein 5 (FABP5)). However, these biomarkers must first be adequately quantified in saliva, and the diagnostic utility of these tests must be established before they can be applied in routine clinical practice [13]. This includes determining cut-off values to assess their sensitivity (Se) and specificity (Sp) [14].

Saliva is increasingly used due to its advantages [15], including easy collection, non-invasive sampling, minimal training required, and reduced stress for collection in farm animals such as horses [16]. Therefore, being able to quantify biomarkers in saliva would be very advantageous to establish the health status of horses in clinical conditions, in a rapid and non-invasive way.

Considering that the pathogenesis of gastric ulcer involves activation of inflammatory, immune, and other defence systems [12,17], an increase in biomarker concentrations of these systems is expected. Therefore, if an optimal selection of markers could be accurately measured in the saliva of horses, we would be witnessing a significant advancement in the detection of gastric ulcers. So, including the right salivary biomarkers in an analytical profile would add important information to detect EGUS and hopefully classify the type of disease by differentiating between ESGD and EGGD.

Therefore, and based on the hypothesis that the five salivary markers (namely IL-1F5, PIP, CA-VI, serotransferrin, and albumin), which in a previous proteomic study [12] in horses were up-regulated after experimentally inducing gastric ulcers, would follow the same behaviour under clinical conditions we followed two basic premises. One, that any assay to measure an analyte must be validated to guarantee its usefulness, and two, that in order to be able to apply in clinical practice a screening with these markers it must be assumed that they have sufficient capacity to differentiate non EGUS horses from clinical EGUS horses. The objectives of this work are, firstly, to optimise assays for the proper quantification of the levels of five gastric ulcer biomarkers in saliva samples. Secondly, to quantify the concentrations of the five biomarkers in samples from horses in different clinical conditions. And thirdly, to evaluate their diagnostic power for its application in clinical practice.

## 2. Materials and Methods

### 2.1. Animals and Samples

#### 2.1.1. Animals

The minimal sample size required for the analysis of the selected biomarkers was calculated based on previous experimental data [12]. Considering the lower effect size of 2.11, a power of 95% and an alpha error of 0.05, the minimum number of horses needed in each group should be 8. Considering that there might be some variability under clinical conditions, the minimal sample size in our study was considered as 2 times the required sample size in experimental conditions, specifically 16 horses per group.

Three groups of horses were enrolled in our clinical assay:

(1) No EGUS horses: 22 adult horses, mares and geldings, aged between 3 and 20 years, from Lexington (KY, USA) with no clinical signs or suspicion of gastric ulcers or other disease according to veterinary clinical examination. In addition, the gastric ulcers were ruled out by gastroscopy.
(2) EGUS non-clinical horses: 28 mares and geldings, between 3 and 14 years, from Lexington (KY, USA), without apparent clinical signs of EGUS or changes in behaviour or performance but with gastric lesions on gastroscopy, were enrolled. A total of 6 horses were classified with ESGD, 11 with EGGD, and 11 horses with both (ESGD + EGGD).
(3) EGUS clinical horses: This consisted of 37 horses, including mares and geldings aged 3 to 18 years old, with clinical signs of EGUS, sourced from the Hagyard Equine Medical Institute (Lexington, KY, USA), where the gastroscopies were performed to confirm the gastric lesions. Among the 37 animals, 9 horses were diagnosed with ESGD, 8 horses with EGGD, and 11 with ESGD + EGGD. The remaining 9 horses showed no evident gastroscopic lesions and were excluded from the study.

Specialised equine veterinarians carried out physical examination, sampling, and gastroscopy of the animals. Squamous lesions were scored following the grading scale of Sykes and Jokisalo [18], while EGGD was exclusively classified as positive or negative considering the presence or absence of any mucosal lesion without grading. Details about scoring for ESGD and lesions for EGGD are described in the Supplementary Table S1.

The horses belong to a feed supplementation study conducted at Clemson University (Clemson, SC, USA) where the animal procedures were approved (Institutional Animal Care and Use Committee of Clemson University guidelines (AUP#2020-013)) and followed the European Directive 2010/63/EU on the protection of animals used for scientific purposes.

#### 2.1.2. Saliva Samples

Saliva was individually collected with a sponge cut into pieces of approximately 3 × 2 × 4 cm (Koronis^®^, Madrid, Spain) inserted through the bars of each horse’s mouth and chewed until saturated with saliva (for about 90 s) during the physical examination and just before the gastroscopy. The sponges were placed in Salivette^®^ tubes (Sarstedt, Numbrecht, Germany), transported to the laboratory at 2–8 °C, and centrifuged at 3200 rpm for 15 min. The samples were stored in 1.5 mL tubes at −80 °C.

### 2.2. Quantification of Salivary Biomarkers

The quantification of IL1-F5, PIP, and CA-VI concentrations in saliva samples was performed using commercial ELISA kits (Invitrogen™ EH264RB (Invitrogen, Carlsbad, CA, USA), Cusabio™ CSB-E15980h (Cusabio, Houston, TX, USA), and Abcam™ ab275899 (Abcam, Waltham, MA, USA), respectively). The kits were used according to the manufacturer’s instructions and optimised for horse saliva. The saliva dilutions used in the commercial kits were 1:3 and 1:4 for IL1-F5 and PIP, respectively, while no dilution was required for CA-VI. Calibration curves for IL1-F5 ranged from 0.069 ng/mL to 50 ng/mL, for PIP from 0.8 ng/mL to 40 ng/mL and for CA-VI from 15.56 pg/mL to 1000 pg/mL.

Non-competitive sandwich ELISAs were developed for serotransferrin and albumin quantification. For the serotransferrin, antibodies against the horse protein were used, specifically a sheep polyclonal antibody (Bethyl™ A70-110A, Bethyl laboratories, Mongomery, TX, USA) at 500 ng/mL as a capture antibody, and a sheep polyclonal antibody (Bethyl A70-110A) labelled with biotin (EZ-Link™ Sulfo NHS-LC-LC-Biotin, 21338, Thermo Scientific, Rockford, IL, USA) at 1000 ng/mL as a detection antibody. The calibration curve was constructed with purified serotransferrin from horse’s serum at a range from 2.7 ng/mL to 175 ng/mL (Supplementary Figure S1a). A salivary 1:64 dilution was used. The ELISA protocol consisted of coating the microplates with 100 µL of the capture antibody overnight, followed by blocking with 300 µL of PBS-0.1% Tween 20 (PBST) and 5% skim milk overnight. After one hour of incubation with biological samples or calibrators, the microplates were incubated for one hour with the detection antibody. A horseradish peroxidase-labelled streptavidin diluted 1:100 (Ultra Streptavidin-HRP™, Thermo Scientific, Rockford, IL, USA) was incubated for one hour, and an ABTS substrate was used for signal development. All incubation steps were followed by 4 times wash with 300 µL of PBS-0.1% Tween 20. Absorbance was measured after 25 min at 405 nm in a microplate spectrophotometer (Spectro Star Nano, BMG Labtech, Ortenberg, Germany).

The albumin ELISA assay was performed using 500 ng/mL of a sheep anti-horse albumin antibody (Bethyl™ A70-122A, Bethyl Laboratories, Montgomery, TX, USA) as the capture antibody and an HRP-conjugated sheep anti-horse albumin antibody (Bethyl™ A70-122P, Bethyl Laboratories, Montgomery, TX, USA) as the detection antibody (at 2000 ng/mL). The calibration curve was constructed with purified albumin from horse serum at a concentration range from 3.12 ng/mL to 200 ng/mL (Supplementary Figure S1b). The saliva samples were diluted 1:6000. ABTS substrate was used for signal development, and the absorbance was measured after 5 min at 405 nm in a microplate spectrophotometer (Spectro Star Nano, BMG Labtech, Ortenberg, Germany).

All protein levels were quantified by interpolation of the absorbances into a 4-parameter curve fit, using GraphPad statistical software (GraphPad Prism version 10.2.0, Boston, MA, USA, www.graphpad.com).

### 2.3. Analytical Validation of the Assays

A basic analytical validation of all ELISA assays was performed, including the calculation of the precision, accuracy, and limit of detection.

To analyse precision, the coefficient of variation (CV) of the measurement of four salivary pooled samples (two of high and two of low concentrations of each biomarker) six times in the same analytical run for the intra-assay precision, and five times in different analytical runs for the inter-assay precision, were calculated as the (mean/standard deviation) × 100.

Linearity under dilution of two samples in four serial dilutions was used to assess accuracy. Curves relating the concentration of each analyte measured to the theoretical concentration expected for each dilution were constructed, and the regression coefficient between expected and measured concentrations was calculated.

The limit of detection was calculated by the following formula: mean blank + 2 × SD, where the mean blank is the mean value obtained when measuring a blank ten times, and the SD is the standard deviation of those determinations [19].

### 2.4. Statistical Analysis

#### 2.4.1. Analytical Validation of Assays

Microsoft Excel was used to determine intra- and inter-assay coefficients of variation (CV) for each of the biomarkers. The linearity under dilution was studied with a linear regression analysis using GraphPad software.

#### 2.4.2. Clinical Validation

Initially, normality and homoscedasticity of the data were determined using the Shapiro–Wilk and Bartlett’s test, respectively. For data that did not meet the homoscedasticity criteria, regardless of whether they were normally distributed, the Welch correction was used. To compare the levels of each analyte between the different groups of horses (No EGUS, EGUS non-clinical, and EGUS clinical), the Brown–Forsythe and Welch ANOVA test was performed. Then, for both EGUS clinical horses and EGUS non-clinical horses, comparisons of the analytes by gastric ulcer type (ESGD, EGGD and ESGD + EGGD) were also established. For these comparisons we followed the same statistical criteria, applying the Brown–Forsythe and Welch ANOVA test in all biomarkers except IL1-F5, where we applied a one-way ANOVA test. Statistical significance was set at *p* < 0.05 for all analyses using GraphPad software.

To calculate the size effect between the concentration of the different parameters in all groups of horses, Cohen’s d was calculated using Jamovi software (version 2.6.26). The interpretation of results was performed according to Cohen’s criteria [20], where values above 0.8 were considered large, between 0.5 and 0.8, medium, and between 0.2 and 0.5, small.

The median ulcers scores and SEM were also set using GraphPad software.

#### 2.4.3. Diagnostic Performance of the Assays

To determine the cut-off values that distinguish the No EGUS group from the EGUS clinical group, ROC analyses were performed for each analyte, using GraphPad software. Each cut-off value is associated with a specificity and sensitivity, and the area under the curve (AUC) of each analyte was also established. AUC values were interpreted according to the guidelines established by Swets [21], where AUC > 0.9 is considered high precision, moderate if AUC range between 0.7 and 0.9 or low if AUC is < 0.7.

## 3. Results

### 3.1. Clinical Signs and Gastric Lesions of the EGUS Horses

The main clinical signs observed in the animals from EGUS clinical, as well as the percentage in which they appeared, are shown in Table 1. The 82.15% of the horses had more than one evident clinical sign.

The overall lesions observed by gastroscopy in the glandular mucosa of animals with EGUS, from both non-clinical and clinical EGUS horses, are shown in Table 2. For further individual details, see Supplementary Table S1.

### 3.2. Analytical Validation of Assays

**Figure 1:**
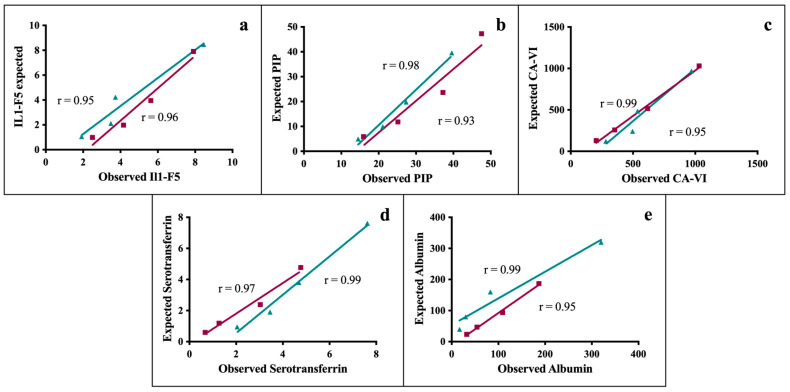
Linear regression lines indicating the accuracy of the assays used for measuring each biomarker in serial dilution of two saliva samples with different concentrations of proteins. The slopes of the regression lines are indicated for each serial dilution. (**a**) IL1-F5, (**b**) PIP, (**c**) CA-VI, (**d**) serotransferrin, and (**e**) albumin.

The ELISA assay used for the quantification of IL1-F5 protein in horse saliva showed a precision of 9.02% (including intra- and inter-assays; details in Table 3). The accuracy showed associations between observed and predicted values of 95–96% (Figure 1a), and a limit of detection of 0.42 ng/mL. The validation of the ELISA kit used to quantify PIP in horse saliva showed a precision (intra and inter-assay) of 12.38% (breakdowns in Table 2), an accuracy exceeding 95% (Figure 1b) and a detection limit of 1.16 ng/mL. The ELISA kit for CA-VI had a detection limit of 19.79 pg/mL, an accuracy of 97% (Figure 1c), and a total precision of 6.79% (details in Table 3). Serotransferrin assay showed a detection limit of 4.68 ng/mL, an accuracy of 98% (Figure 1d), and a total precision of 10.5% (Table 3). The precision of the albumin assay was set at 7.1% (Table 3), the accuracy was 97% (Figure 1e) and the detection limit of 4.34 ng/mL.

### 3.3. Clinical Validation

Descriptive statistics of the quantification performed in the three groups of horses for all parameters are shown in Table 4.

**Figure 2:**
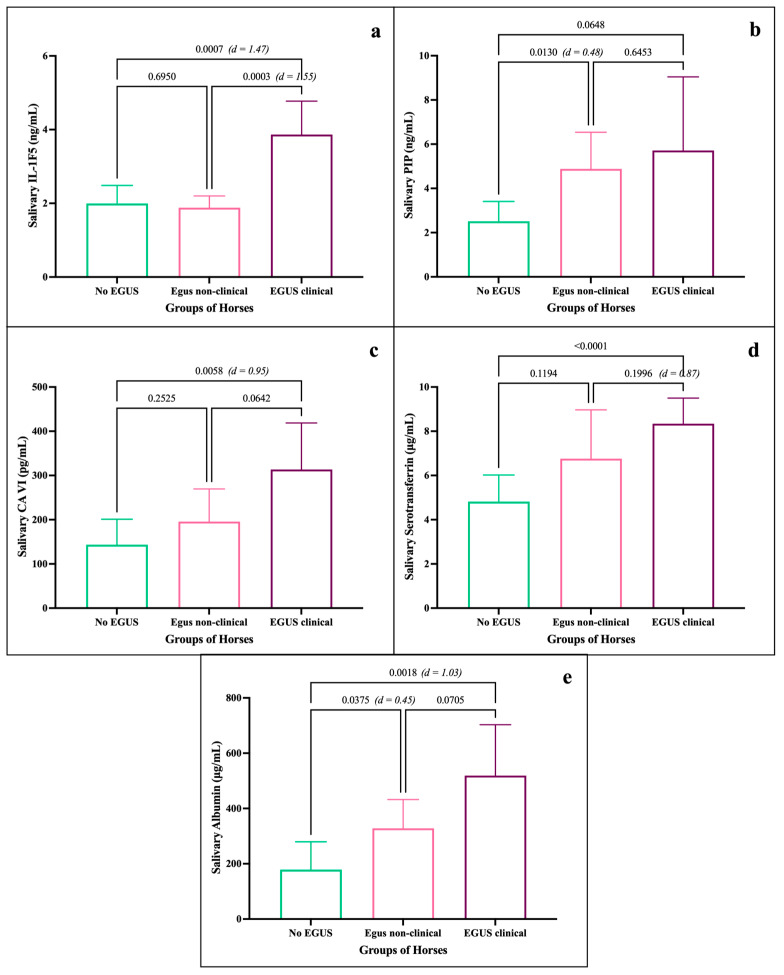
Salivary biomarker concentrations between the different groups of horses: No EGUS (*n* = 22), EGUS non-clinical (*n* = 28) and EGUS clinical (*n* = 28). Mean levels of IL1-F5 (**a**), PIP (**b**), CA-VI (**c**), serotransferrin (**d**), and albumin (**e**), where the error bars represent the standard deviation (SD). The *p*-value is indicated in all comparisons, and the effect size (Cohen d) only when there were statistically significant differences.

Mean concentrations of IL1-F5 showed statistically significant differences between the EGUS clinical group (3.86 ± 1.88 ng/mL) and the other groups, specifically, No EGUS (1.99 ± 1.09 ng/mL), and EGUS non-clinical (1.88 ± 0.81 ng/mL) groups, as shown in Figure 2a. A large size effect was observed between No EGUS and EGUS clinical horses (d = 1.43), exceeded only by the effect size between EGUS non-clinical and EGUS clinical horses (d = 1.55). Mean PIP levels (Figure 2b) reflected statistically significant differences between No EGUS (2.51 ± 1.92 ng/mL) and EGUS non-clinical horses (5.12 ± 4.25 ng/mL). No differences were observed between the PIP levels of the EGUS clinical and No EGUS horses (*p* = 0.06) or with EGUS non-clinical horses (*p* = 0.64). Mean concentrations of CA-VI (Figure 2c) of the EGUS clinical horses (313.57 ± 230.85 pg/mL) showed statistically significant differences with No EGUS horses (143.85 ± 122.86 pg/mL), also showing a large effect size (d = 0.92). The mean serotransferrin concentrations of the EGUS clinical group (8.34 ± 2.92 µg/mL) showed statistically significant differences with the No EGUS group (5.24 ± 3.18 µg/mL), as shown in Figure 2d. A large effect size was observed between both groups (d = 0.87). Albumin analysis (Figure 2e) showed statistically significant differences between the EGUS clinical (519.03 ± 446.02 µg/mL) and No EGUS groups (178.62 ± 202.54 µg/mL), with large effect sizes (d = 1.03), as well as between this latter group and the EGUS non-clinical (328.15 ± 268.36 µg/mL; d = 0.45).

When analysing the ESGD ulcer scores, it was found that in the EGUS non-clinical group the mean was 2.33 (SEM= 0.21) for horses with ESGD and 1.91 (SEM = 0.16) for horses with ESGD + EGGD. On the other hand, in the EGUS clinical group the mean ulcer score was 2.67 (SEM = 0.33) in horses with only ESGD, and 2.36 (SEM = 0.36) when it was present ESGD + EGGD.

**Figure 3:**
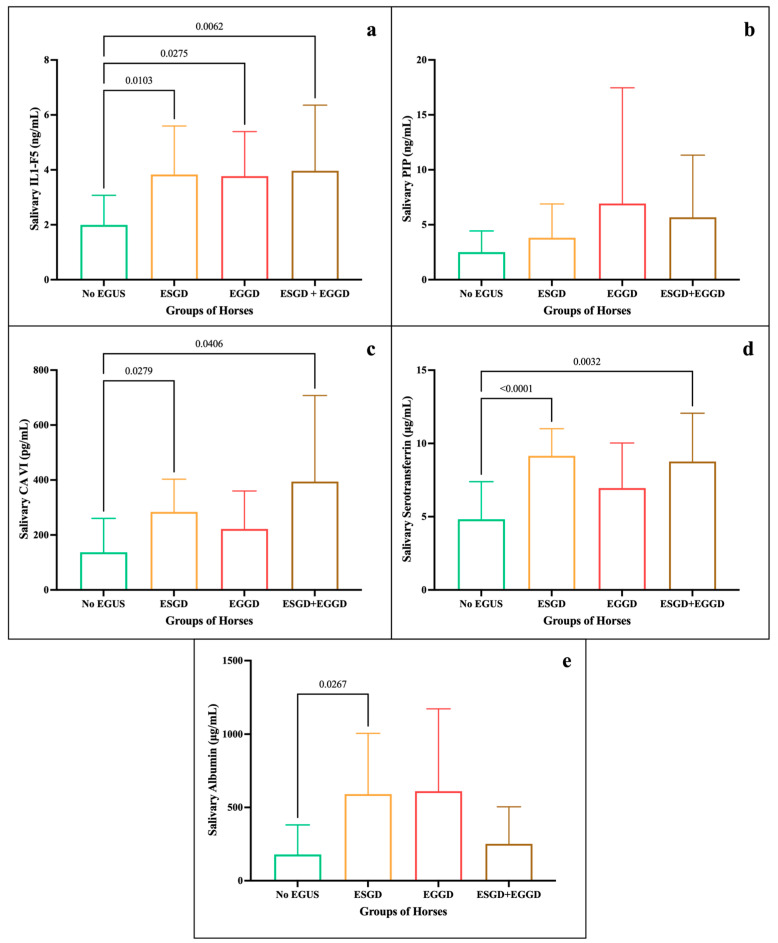
Salivary biomarker concentrations between the different groups of horses: No EGUS (*n* = 22) and EGUS clinical subdivided into ESGD (*n* = 9), EGGD (*n* = 8) and ESGD + EGGD (*n* = 11). Mean levels of IL1-F5 (**a**), PIP (**b**), CA-VI (**c**), serotransferrin (**d**), and albumin (**e**), where the error bars represent the standard deviation (SD). The *p*-value is indicated when there were statistically significant differences.

**Figure 4:**
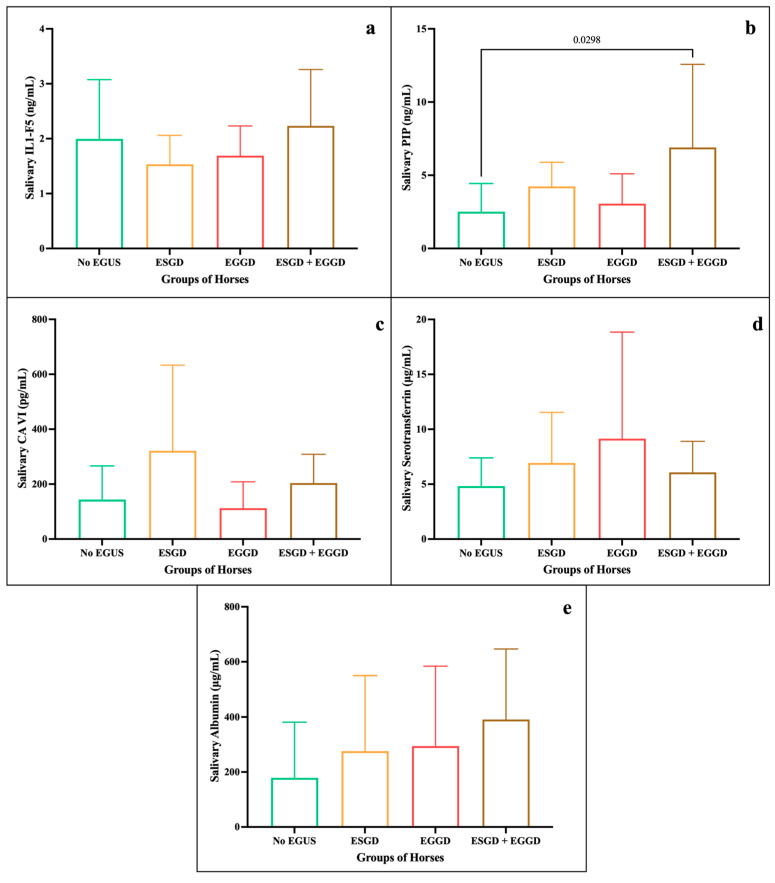
Salivary biomarker concentrations between the different groups of horses: No EGUS (*n* = 22) and EGUS non-clinical subdivided into ESGD (*n* = 6), EGGD (*n* = 11) and ESGD + EGGD (*n* = 11). Mean levels of IL1-F5 (**a**), PIP (**b**), CA-VI (**c**), serotransferrin (**d**), and albumin (**e**), where the error bars represent the standard deviation (SD). The *p*-value is indicated when there were statistically significant differences.

When breaking down the horses with EGUS by type, both in the clinical and non-clinical group, differences in concentration for each biomarker analysed could be observed (descriptive statistics in Supplementary Table S2. For EGUS clinical, the mean levels of IL1-F5 (Figure 3a) were similar in ESGD, EGGD, and ESGD + EGGD (>3.7 ng/mL), with statistically significant differences with No EGUS. In PIP (Figure 3b), no significant differences were seen between the groups; however, levels were highest in EGGD, followed by ESGD + EGGD. Statistically significant higher concentrations of CA-VI (Figure 3c) were seen in horses with ESGD + EGGD, and ESGD (394.3 and 284.2 pg/mL, respectively) in comparison with No EGUS. Similarly, the group of ESGD and ESGD + EGGD showed statistically significant higher levels of serotransferrin (9.15 and 8.76 µg/mL, respectively) in comparison of No EGUS (Figure 3d). However, the albumin only showed statistically significant differences between No EGUS and ESGD. On the other hand, for EGUS non-clinical (Figure 4) highlight that the statistically significant differences were only observed between No EGUS and ESGD + EGGD group for the concentrations of PIP. Neither other salivary biomarkers nor in any EGUS type showed statistical modifications in its concentrations.

### 3.4. Diagnostic Performance

**Figure 5:**
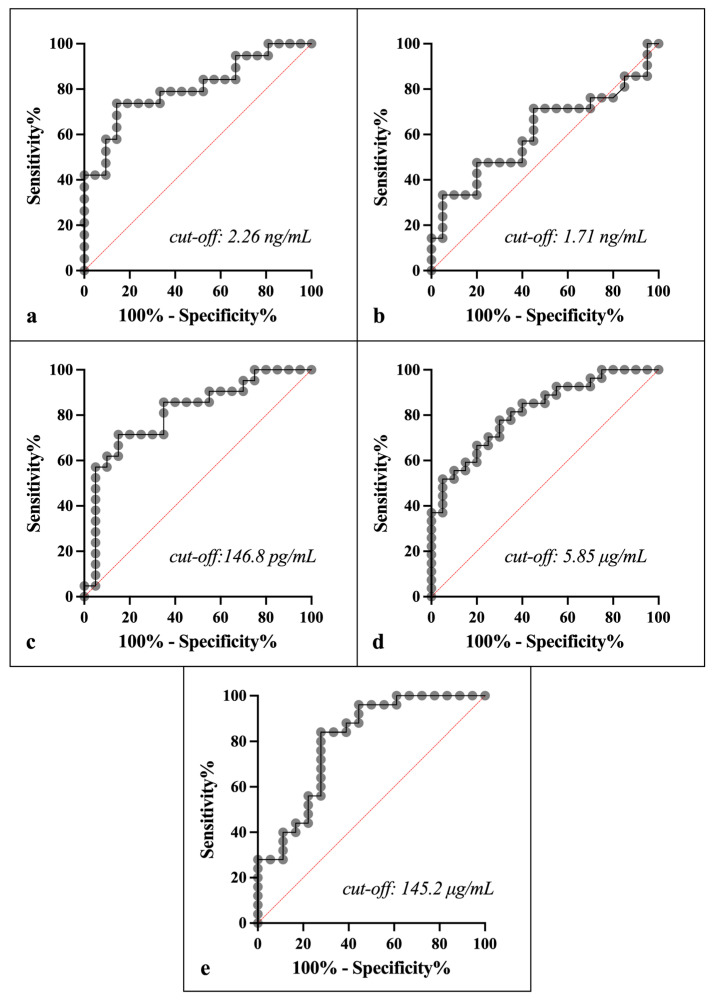
ROC curves and cut-offs of IL1-F5 (**a**), PIP (**b**), CA-VI (**c**), serotransferrin (**d**), and albumin (**e**), between the No EGUS horses and the EGUS clinical horses. The x-axis represents the % of specificity, and the y-axis the % of sensitivity. The cut-off points represent the value with the best pair sensitivity-specificity.

The ROC analyses of IL1-F5, CA-VI, serotransferrin and albumin showed sensitivity and specificity higher than 77 and 65%, respectively, for distinguishing between the No EGUS horses and EGUS clinical horses, with greater AUC in serotransferrin and CA-VI (AUC 0.82 and 0.81, respectively), followed by IL1-F5 and albumin (0.8 each). However, the PIP showed a lower accuracy of 0.61 in distinguishing between the two health conditions (details in Figure 5 and Table 5).

## 4. Discussion

This study represents a first attempt to evaluate the potential use of non-invasive tools for detecting gastric ulceration in horses under clinical conditions by quantifying and subsequently examining the diagnostic value of five salivary biomarkers, specifically IL1-F5, PIP, CA VI, serotransferrin, and albumin.

For that end, commercial human immune-assays have been optimised for the quantification of IL1-F5, PIP, and CA VI, and horse-specific immune-assays have been developed for the measurement of serotransferrin and albumin in horse saliva samples.

The analytical validation of the immuno-assays used to quantify the five analytes in horse saliva showed high precision, accuracy, and good linearity under dilution, and the detection limits allowed quantification with good analytical performance, following general guidelines [22].

We evaluated lesions and the levels of the five salivary biomarkers in No EGUS horses, EGUS non-clinical and EGUS clinical horses to explore as many clinical options as possible with them.

When the scores for squamous mucosal lesions were analysed, animals in the EGUS clinical group had a higher grade than the non-clinical group, and the score was higher when lesions appeared only in ESGD than when they also appeared in the glandular mucosa (ESGD + EGGD) in all cases. Lesions of the glandular mucosa varied in both clinical and non-clinical EGUS, regardless of whether they were isolated (EGGD) or not (ESGD + EGGD). Although fewer hemorrhagic lesions were observed in the clinical group, we believe this could be due to a lack of unanimity in description and highlights the importance of following a more rigorous and uniform descriptive protocol in the future. It would also be necessary to be able to relate the severity of the lesions to the markers.

Our study revealed that the mean concentrations of IL1-F5, CA-VI, serotransferrin and albumin were significantly higher in horses with clinical gastric ulcers compared to No EGUS horses, which is consistent with previous findings in salivary proteomic profiles of horses with induced gastric ulcers [12]. Among these biomarkers, albumin registered the most pronounced differences, with concentration values in EGUS clinical horses nearly 3 times higher than in No EGUS animals. This pattern aligns with the suggestion of the role of an inflammatory response in the variation in albumin levels, as observed in serum from horses with colic [23], next to an increase in serotransferrin levels, which is also consistent with our work. Increased albumin levels have been observed in the saliva due to epithelial barrier dysfunction in oncology patients with chemotherapy-induced gastric lesions [24], as well as in horses undergoing physical exercise [25]. Further studies are needed to clarify their role in equine gastric ulceration. On the other hand, the elevated serotransferrin levels observed in ulcerated horses with clinical signs could be the result of increased salivary synthesis as it has been reported that is partially synthesised in the salivary glands [26], a role as a positive acute phase protein as observed in chickens [27] or other mechanisms that would require further exploration.

The second analyte with the highest increase in its concentration in EGUS clinical horses in comparison to No EGUS animals was CA-VI, with an increase in more than twice the concentration. This increase could be related to the buffering role of the protein protecting the mouth and upper digestive tract against excess acidity described in humans [28].

The increased levels of IL1-F5 in EGUS clinical animals are consistent with its role as an anti-inflammatory cytokine involved in innate immunity and inflammatory diseases [29,30]. Another previous study has reported elevated saliva levels of two inflammatory biomarkers (S100A12 and ADA) in horses with gastric ulcers [17], supporting the suggestion that gastric ulcers have an important inflammatory component. The fact that the concentration of IL1-F5 in the EGUS non-clinical horses is close to that of the No EGUS horses, but significantly lower than that of the EGUS clinical ones, could indicate different states of the inflammatory response that should be further studied.

On the other hand, the levels of the five biomarkers in the horses with EGUS clinical were higher than in the EGUS non-clinical horses, this behaviour could suggest that they may need more time to reach the state of horses with gastric ulcers and clinical signs. Another serum proteomic [31] study also revealed differences in the amount of proteins, studied by electrophoresis, between horses that showed ESGD with and without clinical signs, which gives consistency to our results in EGUS non-clinical horses. In addition, our study also analysed the behaviour of biomarkers in EGUS non-clinical horses compared to No EGUS horses, showing higher levels of PIP and albumin in EGUS non-clinical horses, which suggests that those biomarkers could be used to differentiate between the two conditions and that both biomarkers could be used for an early detection of the disease. Although its functions are not fully established, PIP is involved in the immune response and has been suggested to contribute to the defence against microbial infections [32].

In this study, we have also evaluated the behaviour of the different biomarkers according to the type of EGUS, and the results were different depending on whether the EGUS group was clinical or non-clinical. In EGUS clinical horses, CA-VI and serotransferrin concentrations were significantly higher than in No EGUS horses whenever there were lesions in the squamous mucosa, whereas albumin was only significantly higher if the same lesions were exclusively in the squamous mucosa. However, according to our results, distinguishing between the different types of EGUS appears to be more complicated in the absence of obvious clinical signs. Further studies aimed at differentiating the type of EGUS are therefore needed.

While elevated biomarker levels in ulcerated horses provide valuable insights, clinical efficacy requires careful evaluation of test performance [13]. Therefore, ROC curve analysis was used to establish the threshold values for the five biomarkers to distinguish clean horses from their counterparts with gastric ulcers. ROC curves provide a balance between high sensitivity and high specificity [33]. The analysis of the diagnostic value of the five analytes has been limited to differentiating between No EGUS and EGUS clinical animals, which could help in clinical practice. Based on this, IL1-F5, CA-VI, serotransferrin and albumin showed a moderate accuracy to distinguish between both groups of horses, according to the value of the AUC (around 0.8). This analysis plots sensitivity against specificity for all possible threshold values of the parameter, with the AUC serving as an indicator of the diagnostic accuracy of the parameter [14]. Our results were very close to those obtained in a previous clinical study [17], in which ADA and S100A12 were quantified in horse saliva, and whose ROC analysis also showed moderate accuracy in differentiating healthy horses from those with gastric ulcer (AUC of 0.84 for each).

Hence, our discriminant analysis supports the previously discussed results of clinical validation between the two groups of horses (No EGUS and EGUS clinical) and highlights the potential of four biomarkers (IL1-F5, CA-VI, serotransferrin and albumin) as screening tools, warranting further investigation into their clinical application. Therefore, the combination of these salivary biomarkers in a complete salivary panel, next to other salivary biomarkers such as ADA, S100A12 [17] or even the oxidative stress biomarkers [12,34], may enhance their diagnostic utility in practice to detect animals with gastric ulceration and even to distinguish between the clinical states of the EGUS. Furthermore, in the future we need to further analyse these salivary biomarkers to establish until which extent they would be able to differentiate ESGD from EGGD. This is important in the clinic, in terms of early detection, prevention, or treatment.

## 5. Conclusions

IL1-F5, PIP, CA-VI, serotransferrin and albumin could be quantified in horse saliva samples through ELISA assays, with high precision and accuracy, and showed differences between No EGUS horses and EGUS clinical horses. However, in EGUS non-clinical animals, biomarker increases are only significant in PIP and albumin.

The diagnostic value of four out of five salivary biomarkers, specifically IL1-F5, CA-VI, serotransferrin and albumin, demonstrated moderate accuracy to differentiate No EGUS horses from EGUS clinical horses. These findings represent a promising foundation for the use of salivary biomarkers as analytical tools for helping on the detection of horses with gastric ulcers, highlighting their potential for initial screening tool in clinical conditions.

## 6. Patent

The results of this study are protected under US patent application USPTO 63/718,379.

## Figures and Tables

**Table 1:** Clinical signs observed in EGUS clinical horses (*n* = 28) and frequency of occurrence.

Main Clinical Signs	%
Girthiness	57.14
Bad attitude	42.85
Behaviour change	17.85
Weigh loss	17.85
Picky eater	14.28
Decreased performance	10.71
Unwilling to work	10.71
Decreased appetite	10.71
Stereotypies (such as cribbing, teeth grinding)	7.14
Aggressive	7.14
Poor coat quality	7.14
Colic	3.57
Touch the right side	3.57
Allergies related to food	3.57

**Table 2:** Lesions observed in glandular mucosa in EGUS non-clinical (*n* = 21) and EGUS clinical (*n* = 17), and frequency of occurrence. Data are expressed as percentage (%) divided between horses with EGGD and horses with ESGD + EGGD.

Lesions		Frequency (%) in EGGD	Frequency (%) in ESGD + EGGD
EGUS non-clinical			
Extension	Focal	23.8	28.6
	Multifocal	23.8	23.8
Shape	Flat	33.3	47.6
	Raised	14.3	4.8
Type	Hemorrhagic	4.8	28.6
	Hyperemic	14.3	19
	Fibrinous	19	
	Erosive	4.8	
	Several lesions		9.5
EGUS clinical			
Extension	Focal	11.8	17.6
	Multifocal	23.5	23.5
	Difuse	5.9	5.9
	Linear	5.9	5.9
Shape	Flat	11.8	47
	Raised	29.4	11.8
Type	Hemorrhagic	11.8	5.9
	Hyperemic	11.8	11.8
	Fibrinous	17.6	11.8
	Undescribed	11.8	17.5

**Table 3:** Precision of the ELISA developed for biomarker quantification in saliva samples of horses.

		Intra-Assay			Inter-Assay			
		*Mean*	*SD*	*CV*	*Mean*	*SD*	*CV*	*Total CV*
IL1-F5 (ng/mL)	High pool 1	9.83	0.79	8.02	8.03	0.97	12.10	
	High pool 2	10.85	0.84	7.72	7.63	1.15	15.13	
	Low pool 1	0.77	0.02	2.50	3.29	0.26	7.92	
	Low pool 2	1.31	0.06	4.91	4.88	0.68	13.88	
	*Total*	*5.69*	*0.43*	***5.79***	*5.96*	*0.77*	***12.26***	***9.02***
PIP (ng/mL)	High pool 1	129.09	10.43	8.08	150.88	14.73	9.77	
	High pool 2	110.63	15.51	14.02	158.73	16.34	10.29	
	Low pool 1	4.80	0.67	13.96	3.41	0.47	13.91	
	Low pool 2	8.30	10.08	1.48	5.43	0.78	14.34	
	*Total*	*63.65*	*7.02*	***12.68***	79.62	8.08	***12.08***	***12.38***
CA-VI (pg/mL)	High pool 1	358.31	28.02	7.82	385.67	37.43	9.70	
	High pool 2	379.37	13.57	3.58	418.89	27.4	6.54	
	Low pool 1	91.78	5.60	6.11	90.35	3.86	4.27	
	Low pool 2	95.59	4.66	4.88	98.51	11.33	11.51	
	*Total*	*93.49*	*88.40*	***5.59***	*248.35*	*20.00*	***8.00***	***6.79***
Serotransferrin (µg/mL)	High pool 1	4.74	0.51	10.83	6.876	0.718	10.44	
	High pool 2	4.41	0.33	7.572	5.223	0.562	10.76	
	Low pool 1	2.19	0.202	9.189	2.106	0.237	11.26	
	Low pool 2	1.86	0.19	10.63	1.923	0.234	12.16	
	*Total*	*3.303*	*0.31*	***9.56***	*4.032*	*0.438*	***11.16***	***10.36***
Albumin (µg/mL)	High pool 1	270.51	17.81	6.58	256.97	18.48	7.19	
	High pool 2	278.63	6.81	2.44	270.60	17.1	6.32	
	Low pool 1	83.25	6.78	8.14	81.74	8.75	10.71	
	Low pool 2	75.66	6.34	8.38	81.71	5.71	6.99	
	*Total*	*177.01*	*9.43*	***6.39***	*172.75*	*12.51*	***7.80***	***7.09***

SD. Standard deviation; CV. Coefficient of variation (%).

**Table 4:** Descriptive statistics of the saliva concentrations of the five biomarkers in different groups of horses.

Group	*n*	Mean	SD	Range Values
IL1-F5 (ng/mL)				
No EGUS	21	1.99	1.08	0.36–4.51
EGUS non-clinical	27	1.88	0.81	0.53–3.58
EGUS clinical	19	3.86	1.88	1.15–8.50
PIP (ng/mL)				
No EGUS	20	2.51	1.92	0.54–8.53
EGUS non-clinical	25	5.12	4.25	0.64–18.71
EGUS clinical	21	5.71	7.31	0.76–25.75
CA-VI (pg/mL)				
No EGUS	16	174.49	150.66	4.89–565.5
EGUS non-clinical	28	181.92	182.76	33.91–781.30
EGUS clinical	21	313.57	230.85	63.71–1149
Serotransferrin (μg/mL)				
No EGUS	21	4.82	2.57	0.94–9.84
EGUS non-clinical	27	6.76	5.58	1.46–21.56
EGUS clinical	27	8.34	2.92	2.99–12.37
Albumin (μg/mL)				
No EGUS	18	178.62	202.54	14.24–624.30
EGUS non-clinical	28	328.15	268.36	26.40–846.30
EGUS clinical	25	519.03	446.02	64.17–1694

*n*. Number of horses; Range values. Minimum to maximum values.

**Table 5:** ROC analysis results for biomarker concentrations in saliva samples to differentiate clean horses from horses with clinical gastric ulcers.

Biomarkers	Cut-Off Value	Se (%)	Sp (%)	AUC	95% CI	Likelihood Ratio
IL1-F5 (ng/mL)	2.26	78.95	66.67	0.80	0.66 to 0.94	2.37
PIP (ng/mL)	1.71	71.43	55	0.61	0.43 to 0.79	1.59
CA-VI (pg/mL)	146.80	85.71	65	0.81	0.67 to 0.95	2.45
Serotransferrin (μg/mL)	5.85	77.78	70	0.82	0.69 to 0.93	2.59
Albumin (μg/mL)	145.20	84	72.22	0.80	0.66 to 0.94	3.02

AUC (area under curve); Se (Sensitivity); Sp (Specificity); CI (confidence interval).

## Data Availability

Dataset available on request from the authors.

## Supplementary Materials

The following supporting information can be downloaded at: https://www.mdpi.com/article/10.3390/ani15152251/s1. Figure S1. Calibration curves of the ELISA assays: serotransferrin (**a**), and albumin (**b**), where the y-axis is the corrected absorbance, and the x-axis is the extrapolated concentration of the analyte. Table S1. Detail of the ulcerative lesions found in the different types of EGUS (ESGD, EGGD, y ESGD + EGGD), both in EGUS clinical and non-clinical horses. Scoring of gastric ulcers in ESGD and description of lesions in EGGD. Table S2. Descriptive statistics of the five biomarkers in EGUS clinical and EGUS non-clinical, that consisted of the analysis of saliva samples from No EGUS horses and EGUS horses, the latter subdivided into equine squamous gastric disease (ESGD), equine glandular gastric disease (EGGD) and horses with both subtypes (ESGD + EGGD).

## Abbreviations

The following abbreviations are used in this manuscript:

EGUS	Equine Gastric Ulcer Syndrome
ESGD	Equine Squamous Gastric Disease
EGGD	Equine Glandular Gastric Disease
AUC	Area under curve
CV	Coefficient of variation
IL	Interleukin
PIP	Prolactin inducible protein
AAT	Alpha-1 antitrypsin
ApoA4	Apolipoprotein-A4
CA-VI	Carbonic anhydrase VI
FABP5	Fatty acid-binding protein 5
Se	Sensitivity
Sp	Specificity
PBS	Phosphate-Buffered Saline
SD	Standard deviation
ROC	Receiver Operating Characteristic
