# Supplementary material for: Assessment of Salivary Biomarkers of Gastric Ulcer in Horses from a Clinical Perspective
Source: Animals (Basel). 2025 Jul 31;15(15):2251. doi: 10.3390/ani15152251 (PMC12345524; doi:10.3390/ani15152251)
Supplement: Supplementary file 1 [file animals-15-02251-s001.zip › Table S1.pdf]

Table S1. Details of the ulcerative lesions found in the different types of EGUS (ESGD, EGGD, y ESGD + EGGD), both in EGUS clinical and non-clinical horses. Scoring of gastric ulcers in ESGD and description of lesions in EGGD.

	ESGD	EGGD	ESGD score	Extent of EGGD lesion	Shape of EGGD lesion	Type of EGGD lesion	EGGD location lesion
EGUS non-clinical							
1	x	x	2	focal	flat	hemorrhagic	antrum
2	x	x	2	focal	flat	fibrinous + hemorrhagic	
3	x	x	2	focal	flat	hemorrhagic	antrum
4	x	x	2	multifocal	flat	hemorrhagic	pylorus
5	x	x	2	multifocal	raised	hyperemic	antrum
6		x					
7		x		multifocal	flat	fibrinous	pylorus
8	x		3				
9	x		2				
10	x		2				
11	x		2				
12	x	x	2	focal	flat	hemorrhagic	antrum
13		x		focal	flat	hyperemic	antrum
14		x		multifocal	flat	hyperemic	antrum
15		x		focal	raised	hyperemic	antrum
16	x		2				
17		x		focal	flat	hemorrhagic	antrum
18		x		focal	raised	fibrinous	antrum
19		x		focal	raised	fibrinous	antrum
20		x		multifocal	flat	fibrinous	antrum
21	x	x	1	focal	flat	hemorrhagic	antrum
22	x	x	2	multifocal	flat	hemorrhagic	antrum
23	x	x	3	multifocal	flat	hyperemic	antrum

24	x	x	2	focal	flat	hyperemic	antrum
25	x	x	1	multifocal	flat	hyperemic	antrum
26	x		3				
27		x		multifocal	flat	erosive	antrum
28		x		multifocal	flat	erosive + hyperemic + fibrinous	
EGUS clinical							
1	x		3				
2	x	x	3	focal	flat	fibrinous	pylorus
3		x		lineal	raised	hyperemic	pylorus
4		x		multifocal	raised	fibrinous	pylorus
5	x	x	1	multifocal	flat	hemorrhagic	pylorus
6	x		3				
7		x		multifocal	flat	hemorrhagic	pylorus + fundus
8	x		3				
9	x	x	1				
10	x		4				
11	x	x	2	multifocal	flat	hyperemic	pylorus
12	x		1				
13	x	x	1				
14		x		multifocal	raised	fibrinous	pylorus
15	x		1				
16	x	x	4	lineal	flat	fibrinous	pylorus
17	x	x	3	multifocal	flat	hyperemic	pylorus
18		x		focal	raised		pylorus
19	x		3				
20		x		multifocal	flat	hemorrhagic	pylorus
21	x		3				

22		x		diffuse		hyperemic	pylorus
23		x		focal	raised		pylorus
24	x	x	3	multifocal	flat	hemorrhagic	pylorus
25	x		3				
26	x	x	4	diffuse	flat		pylorus
27	x	x	3	focal	raised		pylorus
28	x	x	1	focal	flat	fibrinous	pylorus
