# Supplementary material for: Assessment of Salivary Biomarkers of Gastric Ulcer in Horses from a Clinical Perspective
Source: Animals (Basel). 2025 Jul 31;15(15):2251. doi: 10.3390/ani15152251 (PMC12345524; doi:10.3390/ani15152251)
Supplement: Supplementary file 1 [file animals-15-02251-s001.zip › Table S2.pdf]

Table S2. Descriptive statistics of the five biomarkers in EGUS clinical and EGUS non-clinical, that consisted of the analysis of saliva samples from No EGUS horses and EGUS horses, the latter subdivided into equine squasmotic gastric disease (ESGD), equine glandular gastric disease (EGGD) and horses with both subtypes (ESGD + EGGD).

**IL1-F5 (ng/mL)**							
	**No EGUS**	**EGUS clinical**			**EGUS non-clinical**		
		ESGD	EGGD	ESGD+ EGGD	ESGD	EGGD	ESGD+ EGGD
Mean	1.99	3.83	3.77	3.97	1.53	1.69	2.23
SD	1.08	1.77	1.62	2.39	0.53	0.54	1.02
Minimum	0.36	1.58	1.67	1.15	0.90	1.01	0.53
Maximum	1.25	1.89	2.19	2.38	2.24	2.95	3.58
**PIP (ng/mL)**							
	**No EGUS**	**EGUS clinical**			**EGUS non-clinical**		
		ESGD	EGGD	ESGD+ EGGD	ESGD	EGGD	ESGD+ EGGD
Mean	2.51	3.81	6.94	5.68	4.24	3.06	6.90
SD	1.92	3.08	10.53	5.65	1.65	2.04	5.68
Minimum	0.54	1.11	0.76	1.07	1.34	0.79	0.64
Maximum	8.5	7.62	25.75	19.05	6.06	7.11	18.71
**CA-VI (pg/mL)**							
	**No EGUS**	**EGUS clinical**			**EGUS non-clinical**		
		ESGD	EGGD	ESGD+ EGGD	ESGD	EGGD	ESGD+ EGGD
Mean	136.80	284.20	221.90	394.30	321.30	112.50	203.70
SD	123.50	118.80	138.0	313.40	311.80	95.79	104.90
Minimum	4.49	63.71	78.97	151.0	62.80	33.91	42.51
Maximum	565.50	412.0	447.30	1149	781.30	350.60	350.0
**Serotransferrin (µg/mL)**							
	**No EGUS**	**EGUS clinical**			**EGUS non-clinical**		
		ESGD	EGGD	ESGD+ EGGD	ESGD	EGGD	ESGD+ EGGD
Mean	4.82	9.15	6.95	8.765	6.93	9.14	6.07
SD	2.57	1.85	3.08	3.2	4.61	9.71	2.83
Minimum	0.94	5.92	2.990	3.6	3.310	1.46	3.49
Maximum	9.84	11.88	12.07	12.37	14.93	26.40	12.84
**Albumin (µg/mL)**							
	**No EGUS**	**EGUS clinical**			**EGUS non-clinical**		
		ESGD	EGGD	ESGD+ EGGD	ESGD	EGGD	ESGD+ EGGD
Mean	178.60	590.90	611.0	251.30	275.60	294.0	390.90
SD	202.50	414.80	561.90	253.20	274.50	289.90	255.80
Minimum	14.24	256.50	98.93	12.37	82.35	26.40	71.15
Maximum	624.30	1535	1694	855.50	743.90	763.10	846.30
