# Supplementary figures and images for: Assessment of Salivary Biomarkers of Gastric Ulcer in Horses from a Clinical Perspective
Source: Animals (Basel). 2025 Jul 31;15(15):2251. doi: 10.3390/ani15152251 (PMC12345524; doi:10.3390/ani15152251)

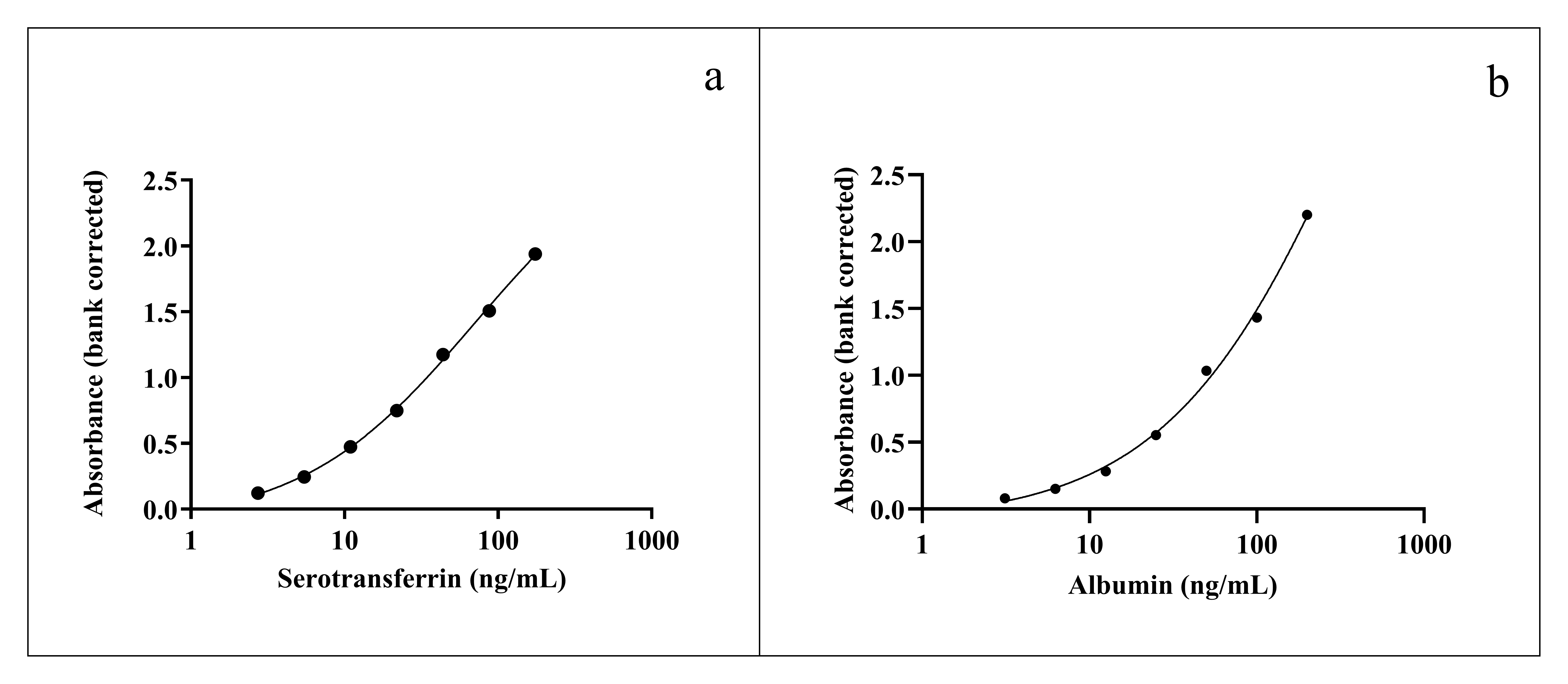

Supplement: Supplementary file 1 [file animals-15-02251-s001.zip › Figure S1.tif]
